# Non-uniform Label Smoothing for Diabetic Retinopathy Grading from Retinal Fundus Images with Deep Neural Networks

**DOI:** 10.1167/tvst.9.2.34

**Published:** 2020-06-30

**Authors:** Adrian Galdran, Jihed Chelbi, Riadh Kobi, José Dolz, Hervé Lombaert, Ismail ben Ayed, Hadi Chakor

**Affiliations:** 1 École de technologie supérieure de Montréal, Montreal, Quebec, Canada; 2 University of Bournemouth, Poole, UK; 3 Diagnos INC, Brossard, Quebec, Canada

**Keywords:** diabetic retinopathy grading, retinal image analysis, label smoothing, deep learning

## Abstract

**Purpose:**

Introducing a new technique to improve deep learning (DL) models designed for automatic grading of diabetic retinopathy (DR) from retinal fundus images by enhancing predictions’ consistency.

**Methods:**

A convolutional neural network (CNN) was optimized in three different manners to predict DR grade from eye fundus images. The optimization criteria were (1) the standard cross-entropy (CE) loss; (2) CE supplemented with label smoothing (LS), a regularization approach widely employed in computer vision tasks; and (3) our proposed non-uniform label smoothing (N-ULS), a modification of LS that models the underlying structure of expert annotations.

**Results:**

Performance was measured in terms of quadratic-weighted κ score (quad-κ) and average area under the receiver operating curve (AUROC), as well as with suitable metrics for analyzing diagnostic consistency, like weighted precision, recall, and F1 score, or Matthews correlation coefficient. While LS generally harmed the performance of the CNN, N-ULS statistically significantly improved performance with respect to CE in terms quad-κ score (73.17 vs. 77.69, *P* < 0.025), without any performance decrease in average AUROC. N-ULS achieved this while simultaneously increasing performance for all other analyzed metrics.

**Conclusions:**

For extending standard modeling approaches from DR detection to the more complex task of DR grading, it is essential to consider the underlying structure of expert annotations. The approach introduced in this article can be easily implemented in conjunction with deep neural networks to increase their consistency without sacrificing per-class performance.

**Translational Relevance:**

A straightforward modification of current standard training practices of CNNs can substantially improve consistency in DR grading, better modeling expert annotations and human variability.

## Introduction

Diabetes is considered a global eye health issue, with a steadily increasing worldwide prevalence that is estimated to reach 629 million individuals by 2045.^1^ Diabetic retinopathy (DR) is a diabetes complication affecting eyes, caused by damage to blood vessels within the retina. DR manifests early signs in the form of swelling microaneurysms breaking small vessels and releasing blood and fluid into the retina. Latest DR stages are characterized by the appearance of advanced signs like the proliferation of new, abnormally fragile blood vessels, potentially leading to retinal detachment and eventually permanent sight loss.

Retinal images acquired with fundus cameras can reliably capture and depict the above signs, thereby representing an effective diagnostic tool.^2^ For this reason, screening programs designed for early DR diagnosis and treatment have been established in developed countries.^3^ In these programs, retinal specialists examine and grade eye fundus images in order to deliver diagnostic outcomes. However, issues related to the scale and cost of screening programs, together with the increasing need of trained specialists, hinder their introduction in developing countries.^3^^,^^4^

Within this context, in recent years, DR detection from eye fundus images has become a fertile ground of application for the new generation of deep neural networks (DNNs). Powered by higher computing capabilities and the availability of large amounts of data, DNNs have enabled unprecedented predictive accuracy in a wide range of diagnostics tasks.^5^^–^^8^ In the field of ophthalmic image diagnosis, this includes not only applications to DR detection/screening^9^^,^^10^ but also to age-related macular degeneration assessment,^11^^,^^12^ glaucoma detection,^13^^,^^14^ or diabetic macular thickening,^15^ to name a few.

In this article, we focus on the problem of DR grading from eye fundus images. This extends the task of DR detection to a multiclass problem, in which the goal is to precisely predict the severity stage of DR. Grading is a harder task than detection, mainly due to the difficulty in modeling high interobserver variability,^16^^,^^17^ and comparatively fewer works have studied this problem.^17^^,^^18^ In particular, in this article, we are concerned with designing a simple mechanism to increase diagnostic consistency in the predictions of a standard DNN tasked to perform DR grading. We understand consistency as the capacity of a model to produce predictions closer to the true grade in cases when the original prediction is wrong. Let us stress that our goal is to achieve such enhanced consistency without incurring a lower overall predictive accuracy.

## Methods

### Data Set

For this study, color images of the eye fundus were acquired between 2016 and 2018 with a nonmydriatic Centervue DRS camera. Patients’ countries of origin were multiple, including Mexico (84.1%), United States (9.7%), Saudi Arabia (2.4%), India (1.8%), Canada (1.3%), and other countries (0.7%). Images were graded according to the American Association of Ophthalmology protocol, assigning to each photograph one out of five possible disease stages or indicating when the image was ungradable. The resulting private data set was divided into independent train, validation, and test sets in proportions of 75%, 10%, and 15%, respectively. A summary of the information relevant to data statistics is given in Table 1.

**Table 1.** Data Set Summary

No. of Images (Unique Individuals)		46,865 (27,361)		
Age (Mean ± SD)		59.6 ± 14		
Female/Male		17,658/9233		
Characteristic	Total, No. (%)	Training, No.	Validation, No.	Test, No.
No DR	31,447 (67.1)	23,585	3,145	4,717
Mild DR	1264 (2.7)	948	126	190
Moderate DR	6822 (14.6)	5117	682	1,023
Severe DR	230 (0.5)	172	23	35
Proliferative DR	683 (1.5)	512	68	103
Ungradability	6419 (13.7)	—	—	—

### Deep Learning and Label Smoothing Regularization

**Figure 1.**
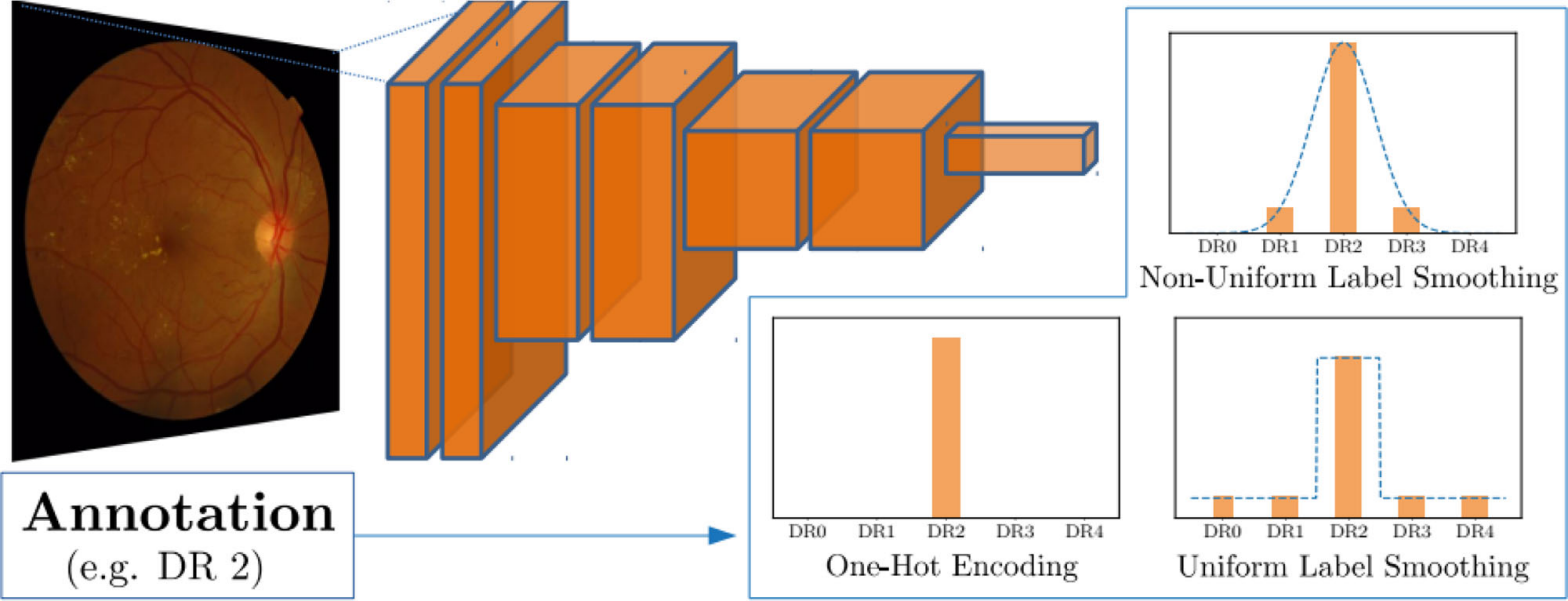
Schematic representation of our proposed label representation.

Deep learning in the context of image diagnosis refers to the optimization of a DNN. DNNs are mathematical models built of sequences of simple operations that transform an input image into a predictive output. In image analysis applications, the most successful models of this kind are convolutional neural networks (CNNs), where such operations are learnable convolutions that repeatedly filter and downsample images. The parameters defining these convolutions are optimized through the iterative observation of annotated data, in our case, of retinal fundus images and associated DR grades, as illustrated in Figure 1. A key component of these systems is the loss function, which drives the optimization process by penalizing wrong predictions and allowing the model to correct its parameters during the learning stage. A typical loss function for image classification tasks is the cross-entropy error (denoted CE in the rest of this article). Let us note that the CE loss does not model any kind of difference between distinct categories. For instance, in the context of DR grading, the penalization imposed by CE to a prediction of DR grade 4 and one of DR grade 3, when the actual disease stage is DR grade 0, will be exactly the same.

In recent years, newer CNN architectures have been introduced in the computer vision field, steadily increasing performance in image classification benchmarks.^19^^,^^20^ Typically, these CNNs contain more learnable parameters, which increases their capacity to process images in different ways, resulting in richer image processing and representation ability. However, models containing more parameters are prone to overfitting, a phenomenon by which the model is exceedingly adapted to the training data and fails to generalize to images that were not used for training. To optimize these complex models with increased robustness and to avoid hurting generalization capability, there is the need for either more training data or stronger regularization techniques. A regularization mechanism is a mathematical addition to the training process that intends to bias the learned model toward simpler solutions, which are expected to generalize better to unseen data.^21^ For instance, a popular regularization approach is to penalize the learned parameters from reaching too large values, which would artificially create overcomplicated decision boundaries.

In this work, we are interested in a simple but much successful approach to regularization, a technique called label smoothing.^19^ This is a method typically applied for multiclass classification tasks, where the standard loss function is the CE error and annotations are represented in a format called *one-hot encoding*, as shown in Figure 1 (center bottom). The simple idea behind label smoothing is to replace these *hard labels* by a smoothed version of them, in which part of their *truth value* is redistributed in a uniform manner among the rest of the labels. Therefore, the new label-smoothed annotations in DR grading would be a weighted average of the hard annotations and a uniform distribution over grades, following the formula *y_k_* = *y_k_*(1 – *a*) + *a*/*N*, for each class *k* in {0, 1, 2, 3, *N* = 4}, as displayed in Figure 1 (right bottom). Parameter *a* specifies the amount of regularization imposed to the network. Label smoothing can contribute to avoiding overconfident models and has been reported to benefit overall accuracy while also increasing learning speed.^19^ Recent research has also shown that label smoothing can improve model calibration and out-of-distribution detection.^22^

### Non-uniform Label Smoothing and Error Consistency

Despite the benefits in accuracy provided by label smoothing in a wide array of computer vision tasks, this technique is not suitable for every problem in a generalistic way. In particular, the smoothing scheme should ideally depend upon any potential underlying structure present on data annotations. While previous works have mainly applied label smoothing on annotations that do not contain such structure, for the case of DR grading, a conceptual distance between disease stages exists. Consequently, we propose to modify the standard label smoothing regularization technique by simply replacing the one-hot encoded annotation *y_k_* by a Gaussian distribution centered at *y_k_* with a decay factor (standard deviation) σ selected in such a way that 95% of the probability mass still falls within its neighboring grades. Mathematically, this would be described as

$$\begin{array}{c} y_{k}={G_{k}}_{,\sigma }\left({y_{k}}_{}\right). \end{array}$$

A graphical representation of the proposed regularization scheme is displayed in Figure 1 (top right). We refer to this modified technique as non-uniform label smoothing (N-ULS) in the remaining.

It should be noted that the “degree of truth” remaining in each label after smoothing using the uniform label smoothing (ULS) and N-ULS approaches is kept constant across all grades. There is, however, some “missing probability” in the corner grades (grades 0 and 4) for the N-ULS case; this could be easily handled by renormalizing the probability mass so that it adds up to 1 in these two grades. However, this would also cause a somehow asymmetric behavior of the N-ULS technique when compared to ULS, as it would place more “degree of truth” in these particular grades. There is necessarily a decision to make in this case, and in this article, we opt for the unnormalized implementation, supported also by our preliminary experimental analysis (we did not observe any noticeable performance difference between the unnormalized and the renormalized strategies).

By implementing N-ULS, we expect to bias the learning of a DR grading CNN toward a model that, when mistaken, produces more consistent errors. This is because N-ULS reflects in a more suitable manner interobserver disagreements: two human graders differing in their opinion will most likely do so by neighboring grades than by faraway ones. N-ULS introduces in this way new information into the optimization process, since when the CNN observes a new data point with associated annotation, it must also learn a notion of the underlying DR grading structure.

### Experimental Design and Evaluation Approach

We first consider a standard CNN, a 50-layer residual network,^20^ which can be regarded as the default computer vision model for most visual perception tasks. We optimize this network by standard backpropagation using the CE loss, since it has been used in most previous works on DR grading.^16^^,^^18^ We also analyze performance when an architecture with more learnable weights (and thus more powerful but also more prone to overfitting) is employed; for this, we experiment with a 101-layer residual network. In all cases, the weights are initialized from a pretraining on the ImageNet data set, and they are iteratively updated by stochastic gradient descent; the error is monitored in the separate validation set, with training being stopped when no further improvement is observed on it. Both networks are trained on the exact same data and under the same baseline configuration three times: first without any label smoothing (CE), second with standard label smoothing (LS), and last with a N-ULS scheme. Learning rate and batch size were set to 0.001 and 8, respectively, for all experiments. All models were trained on a standard NVIDIA 1080Ti GPU card and converged in approximately the same amount of epochs (∼15). A PyTorch implementation of both LS strategies can be found at https://github.com/agaldran/non_uniform_label_smoothing.

To properly assess the performance of the three considered models, the main metric of interest that can capture error consistency as described above is report quadratic-weighted κ score (quad-κ), which is typically used to assess interobserver variability.^17^ Quadratic-weighted κ score is particularly relevant to this work, as it faithfully models the underlying distance in grades present in DR stage classification. We also report the average area under the receiver operating curve (AUROC) in its multiclass extension, after considering each possible class pair.^23^ Finally, we also compute multiclass F1, precision, and recall scores (an average weighted by the support of each class) and analyze correlation between model predictions and expert grades by means of Matthews correlation coefficient (MCC), which has been found useful for assessing imbalanced problems in biomedical applications.^24^

For statistically testing the performance of N-ULS as compared to the other two approaches, human annotations and model predictions in the test set were bootstrapped^25^ (*n* = 1000) in a stratified manner with respect to the relative presence of each grade. Performance differences Δ for each of the above metrics were calculated in each bootstrap and *P* values were computed for testing significance. The statistical significance level was set to α = 0.05, but performance differences were considered statistically significant if *P* < 0.025 due to the Bonferroni correction.^26^

## Results

**Figure 2.**
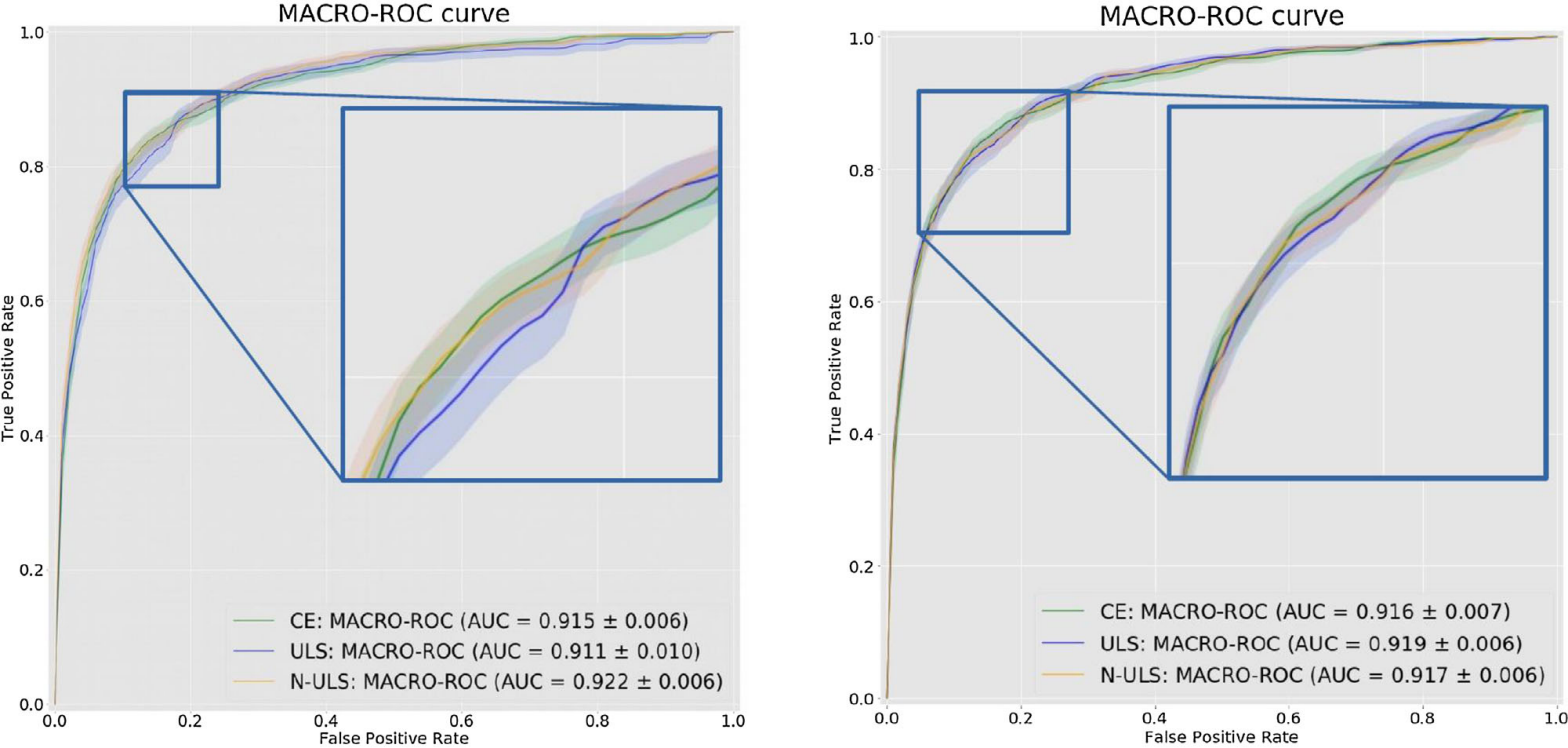
Bootstrapped ROC curve resulting from training a Resnet50 (*left*) and a Resnet101 (*right*) CNN with CE/LS/N-ULS.

Quadratic κ scores, together with the other metrics of interest, and the corresponding statistically significance analysis are reported in Table 2 for the ResNet50 model and in Table 3 for the ResNet101 model. In addition, we show macro-ROC curves for both cases in Figure 2. Regarding the experiments with the ResNet50 architecture, we observed the following:

1) When comparing N-ULS with respect to both CE and LS, the quadratic κ score was substantially improved in +4.52% and +6.43% points, respectively. In either case, the improvements were statistically significant.
2) When comparing N-ULS with respect to LS in terms of average AUROC, the AUROC was statistically significantly better in the comparison against LS (+1.07%), and no statistically significant improvement was observed when comparing to CE.
3) As for the remaining figures of merit, N-ULS statistically significantly outperformed both CE and LS in terms of overall weighted recall, F1, and MCC and surpassed LS in overall precision. N-ULS was never outperformed by any of the compared approaches, and the remaining comparisons were not statistically significant.

**Table 2.** Performance Comparison for the ResNet50 CNN in Terms of Mean Differences in Quadratic-Weighted κ and Other Metrics of Interest, Obtained from 1000 Bootstrap Iterations

Quad-κ			Average AUROC			Weighted F1		
N-ULS	**77.69**		N-ULS	**91.58**		N-ULS	**87.86**	
CE	73.17	**Δ = +4.52 (** ***P*** **<** **0.025)**	CE	91.36	Δ = **+**0.22 (*P* > 0.025)	CE	85.35	**Δ = +2.21 (** ***P* < 0.** **025)**
LS	71.26	**Δ = +6.43 (** ***P* < 0.** **025)**	LS	90.51	**Δ = +1.07 (** ***P* < 0.** **025)**	LS	85.10	**Δ = +2.76 (** ***P* < 0.** **025)**
Weighted Precision			Weighted Recall			MCC		
N-ULS	**87.33**		N-ULS	**89.10**		N-ULS	**68.03**	
CE	86.48	Δ = **+**0.84 (*P* > 0.025)	CE	84.44	**Δ = +4.64 (** ***P* < 0.** **025)**	CE	59.71	**Δ = +8.32 (** ***P* < 0.** **025)**
LS	85.21	**Δ = +2.12 (** ***P* < 0.** **025)**	LS	87.03	**Δ = +2.07 (** ***P* < 0.** **025)**	LS	60.71	**Δ = +7.32 (** ***P* < 0.** **025)**

Statistically significant improvements are marked bold.

**Table 3.** Performance Comparison for tde ResNet101 CNN in Terms of Mean Differences in Quadratic-Weighted κ and Other Metrics of Interest, Obtained from 1000 Bootstrap Iterations

Quad-κ			Average AUROC			Weighted F1		
N-ULS	**77.19**		N-ULS	91.02		N-ULS	**87.91**	
CE	71.98	**Δ = +5.21 (** ***P* < 0.** **025)**	CE	91.48	Δ = –0.46 (*P* > 0.025)	CE	84.76	**Δ = +3.15 (** ***P* < 0.** **025)**
LS	74.52	**Δ = +2.67 (** ***P* < 0.** **025)**	LS	91.26	Δ = –0.24 (*P* > 0.025)	LS	86.78	**Δ = +1.13 (** ***P* < 0.** **025)**
Weighted Precision			Weighted Recall			MCC		
N-ULS	**87.27**		N-ULS	**88.99**		N-ULS	**68.08**	
CE	86.69	Δ = **+**0.58 (*P* > 0.025)	CE	83.64	**Δ = +5.35 (** ***P* < 0.** **025)**	CE	60.90	**Δ = +7.18 (** ***P* < 0.** **025)**
LS	86.39	**Δ = +0.88 (** ***P* < 0.** **025)**	LS	87.97	**Δ = +1.02 (** ***P* < 0.** **025)**	LS	64.37	**Δ = +3.71 (** ***P* < 0.** **025)**

Statistically significant improvements are marked bold.

Concerning the experiments with the ResNet101 model, we note the following observations:

1) N-ULS kept a similar performance in terms of quadratic κ, outperforming again CE and LS (+5.21%, +2.67%). With a more complex architecture, performance of CE was degraded, whereas performance of LS increased.
2) In terms of average AUROC, N-ULS achieved similar performance in this case as the other alternatives CE and LS.
3) Regarding the remaining performance metrics, N-ULS achieved similar performance as in the ResNet50 case, again statistically significantly outperforming CE and LS in terms of recall, F1, and MCC. An overall better performance of LS can be noticed in this case, as well as a general degradation in the performance of CE.

The above observations are discussed and conclusions are drawn in the following section.

## Discussion

The above analysis leads to several conclusions. First, results reported in Table 2 for the ResNet50 case demonstrate that the introduction of standard LS in the training of this network seems to generally harm performance when compared to only using conventional CE. This was also verified in a separate statistical test (not included for brevity), in which we found that using the CE loss in this case resulted in an increase of 1.91 percentage points in the κ score (*P* = 0.046) with respect to using LS. This was not the case for the N-ULS strategy introduced in this article: for all considered metrics, either the performance was significatively increased, or the performance decrease was not statistically significant. Second, and more important, the quadratic κ score was substantially higher for the N-ULS approach, which confirms our hypothesis that the error consistency can be improved by means of a simple domain-specific label smoothing strategy. In both cases, the quadratic-weighted κ score was statistically significantly better when optimizing the network with the N-ULS technique as compared to the other two approaches, verifying the validity of our findings.

Our performance analysis on other metrics for the ResNet50 case clearly demonstrates the benefits of implementing N-ULS over training a CNN with the ordinary CE loss. We also observe for these metrics that LS actually harms performance when compared to CE, but N-ULS recovers much of this performance loss, even rising slightly above CE results. Remarkably, while similar performance levels are obtained by CE and N-ULS in terms of average AUROC, precision, and recall, a model trained with N-ULS significantly outperforms the standard CE version in terms of correlation measurements like F1 score or MCC, aside from the greater quadratic-weighted κ scores observed above.

When repeating the above analysis with the ResNet101 CNN, other interesting consequences arise. First, overall performance when using N-ULS is maintained for every considered metric. Second, the performance of the CE loss without any regularization is considerably degraded in terms of quadratic κ, dropping from 0.732 to 0.719. In contrast, the quadratic κ attained by LS increased from 0.712 to 0.7452. A separate statistical testing of LS versus CE in this case resulted in observing an increase of quadratic κ score equal to 2.54 percentage points (*P* = 0.019). A similar trend can be observed in all the other performance metrics. In general, training a more powerful architecture comes with a greater risk of overfitting, and in this case, LS seems to be successful in reducing this phenomenon, which decreases the performance of the same network trained with standard CE. In any case, N-ULS remains equal or superior to either approaches in terms of every considered performance metric, hinting at its usefulness as a regularization technique independently of the CNN complexity.

The reported experimental results demonstrate that the method introduced in this article for CNN regularization is useful in the context of DR grading. One of the major challenges in extending conventional deep learning–based approaches from DR detection or screening (binary problems) to DR grading (a multiclass scenario) lies in ensuring that the underlying structure of expert annotations is well captured by the network. The approach introduced in this article is a straightforward step toward this goal. As a secondary benefit, N-ULS helps in combating data imbalance (one or several classes having disproportionately less training samples than the other ones), which is a typical obstacle in DR grading (see Table 1). It does so by attaching extra information to each example: the smoothed label corresponding to an image annotated with a particular DR grade conveys the information not only of its own grade but also of which are its neighboring grades.

It is worth mentioning that N-ULS might also be useful as an approach to handle the disproportionate difficulty of correctly classifying fundus images corresponding to the DR1 class. The typical low performance in this category among all existing techniques is explained by the fact that symptoms of mild DR involve the presence of few microaneurysms, which are subtle, easily confused with other visual artifacts, and hard to find even for human experts.^16^ Since algorithms are trained on data sets annotated by human experts, annotations inherit such ambiguity, which is particularly high in this grade of the disease. This is also another motivation for the proposed technique. Since formulating *perfect predictions* is not even possible for experts, it might be more useful to at least make sure that a model formulates instead *reasonable predictions*, in line with the error consistency improvement properties of N-ULS.

N-ULS can be incorporated into existing methodologies that employ standard CE loss functions in order to more appropriately reflect such structure. It is important to remark that the N-ULS regularization scheme is independent of the CNN architecture and could be equally useful in the context of other grading problems like diabetic macular edema prediction. Future work will involve the extension and validation of this technique to other disease grading problems.
